# Domain-specific cognitive impairment 6 months after stroke: The value of early cognitive screening

**DOI:** 10.1177/17474930231205787

**Published:** 2023-10-14

**Authors:** Elise T Milosevich, Margaret J Moore, Sarah T Pendlebury, Nele Demeyere

**Affiliations:** 1 Department of Experimental Psychology, Radcliffe Observatory Quarter, University of Oxford, Oxford, UK; 2 Queensland Brain Institute, University of Queensland, St Lucia, QLD, Australia; 3 Wolfson Centre for Prevention of Stroke and Dementia, Wolfson Building, Nuffield Department of Clinical Neurosciences, University of Oxford, Oxford, UK; 4 NIHR Oxford Biomedical Research Centre and Departments of General Medicine and Geratology, John Radcliffe Hospital, Oxford, UK

**Keywords:** Stroke, stroke teams, neurology, cognitive impairment, vascular dementia, cognitive dysfunction

## Abstract

**Background:**

Cognitive screening following stroke is widely recommended, yet few studies have considered the prognostic value of acute domain-specific function for longer-term cognitive outcome. Identifying which post-stroke cognitive impairments more commonly occur, recover, and persist, and which impairments hold prognostic value, could inform care planning, and resource allocation.

**Aims:**

This study aimed to determine the prevalence of domain-specific impairment acutely and at 6 months, assess the proportion of change in cognitive performance, and examine the prognostic value of acute domain-specific cognitive screening.

**Methods:**

A prospective stroke cohort completed the Oxford Cognitive Screen acutely (⩽2 weeks) and 6 months post-stroke. We determined the prevalence of acute and 6-month domain-specific impairment and proportion of change in performance from acute to 6 months. Hierarchical multivariable regression was used to predict global and domain-specific cognitive impairment at 6 months adjusted for demographic/vascular factors, stroke severity, and lesion volume.

**Results:**

A total of 430 stroke survivors (mean/SD age 73.9/12.5 years, 46.5% female, median/interquartile range (IQR) National Institute of Health Stroke Scale (NIHSS) 5/2–10) completed 6-month follow-up. Acutely, domain-specific impairments were highly prevalent ranging from 26.7% (*n* = 112) in praxis to 46.8% (*n* = 183) in attention. At 6 months, the proportion of domain-specific recovery was highest in praxis (*n* = 73, 71%) and lowest in language (*n* = 89, 46%) and memory (*n* = 82, 48%). Severity of 6-month cognitive impairment was best predicted by the addition of acute cognitive impairment (adj *R*^2^ = 0.298, *p* < 0.0001) over demographic and clinical factors alone (adj *R*^2^ = 0.105, *p* < 0.0001). Acute cognitive function was the strongest predictor of 6-month cognitive performance (*p* < 0.0001). Acute domain-specific impairments in memory (*p* < 0.0001), language (*p* < 0.0001), and praxis (*p* < 0.0001) significantly predicted overall severity of cognitive impairment at 6 months.

**Conclusion:**

Post-stroke cognitive impairment is highly prevalent across all domains acutely, while impairments in language, memory, and attention predominate at 6 months. Early domain-specific screening can provide valuable prognostic information for longer-term cognitive outcomes.

## Introduction

Though the prevalence of post-stroke cognitive impairment varies depending on the timing, assessment, and inclusion criteria, most stroke survivors experience at least one cognitive deficit.^1,2^ Identifying which cognitive impairments more commonly occur, recover, and persist will aid in intervention planning and setting rehabilitation goals.

Early post-stroke cognitive assessment is recommended in clinical guidelines and best practice statements.^3–6^ The Oxford Cognitive Screen (OCS)^7,8^ is a short, domain-specific cognitive screen which compromises between the benefits of global tests and neuropsychological batteries. The OCS offers superior patient inclusivity and sensitivity than brief screens (e.g. the Mini-Mental State Examination (MMSE) and Montreal Cognitive Assessment (MoCA)).^1,9^ It also assesses multiple domains much like a neuropsychological battery, which is in line with clinical guidance. Acute domain impairments are highly prevalent after stroke;^1^ however, the prevalence and trajectories of domain-specific impairment beyond the acute stage are relatively unknown. In addition, while the OCS has recently been shown to provide value information regarding functional outcomes,^10^ it has not been established whether the short screen had prognostic value in determining longer-term cognitive outcome.

Very few studies have considered acute post-stroke cognitive performance in longer-term outcome prediction models, and instead, the focus tends to be on clinical and neuroimaging markers.^11^ A few previous studies have aimed to predict outcomes using brief global screens^12^ (e.g. the MoCA).^13–17^ Acute MoCA scores predict long-term cognitive and functional outcome, and mortality.^13^ However, the MoCA is not always suitable for stroke as it assumes cognitive functions (e.g. speech, vision) are intact. Consequently, common post-stroke impairments (e.g. aphasia, neglect) can confound assessment.^9,12^

One study used a multi-domain neuropsychological battery to investigate the prognostic value of early cognitive assessment^18,19^ and found acute performance to predict 6-month cognitive and functional outcome better than demographic or clinical variables.^19^ These findings support the utility of cognitive data; however, a large neuropsychological battery (>1 h) is typically not feasible in routine acute clinical practice due to time requirements and increased burden on the patient, as well as staff availability and expertise.

This study aimed to use the OCS to (1) determine the prevalence of domain-specific cognitive impairments acutely and at 6 months post-stroke, (2) assess change in function across timepoints, and (3) examine the predictive value of early domain-specific screening. Importantly, this study does not aim to build optimal prognostic models employing all potential predictors (e.g. genetic risk) but instead aims to determine the value added by considering cognitive data. Ultimately, this could lead to practical prognostic models based on routinely collected and easily accessible data, promoting informed and efficient clinical decision-making.

## Methods

This study considered existing data from the OCS-Tablet and OCS-Recovery studies (National Research Ethics Committee (UK), references 14/LO/0648 and 18/SC/0550, in accordance with the Declaration of Helsinki). Patients (⩾18 years old) were included if they could provide written/witnessed informed consent, could concentrate for 20 min, and had sufficient English language comprehension.

### Participants

**Figure 1.**
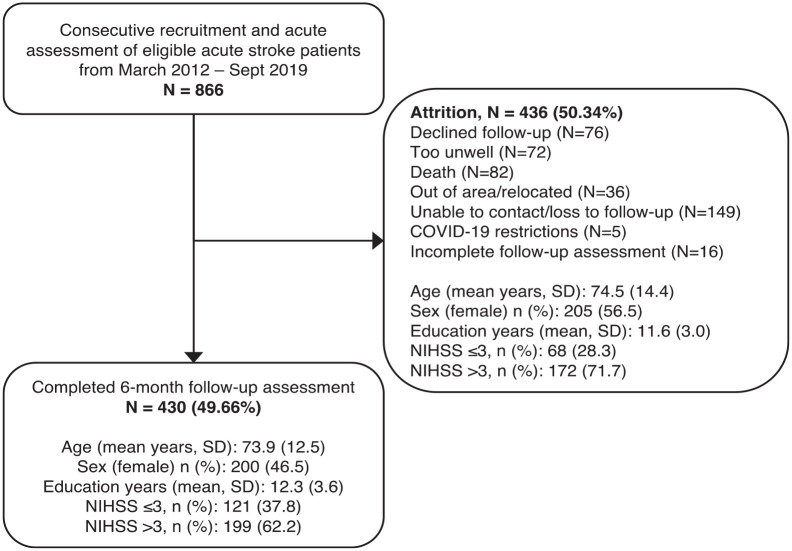
Patient cohort from baseline to 6-month follow-up.

A consecutive sample of acute stroke patients was recruited from the John Radcliffe Hospital, UK, acute stroke unit (2012–2019). In total, 866 patients were assessed acutely (⩽2 weeks), with 430 (49.7%) completing 6-month follow-up (Figure 1).

### Cognitive assessment

The OCS was used for acute and follow-up cognitive screening.^7^ The OCS covers a broad range of cognitive domains and was designed specifically for use in acute stroke taking 15–20 min to complete. Subtests are categorized into six domains: language (picture naming, semantic understanding, and sentence reading), attention (egocentric and allocentric attention; broken hearts test), executive function (trail-making test), memory (orientation, verbal, and episodic memory; delayed recall/recognition), praxis (meaningless gesture imitation), and number processing (calculations and number writing). Each task has a threshold cut-off to indicate impairment based on published normative data.^7,8^ Subtests were binarized into impaired or unimpaired based on normative scores for each subtest. Domains were considered impaired if there is at least one subtest in the domain (e.g. language impaired if reading, naming, or semantics impaired). Subtests range from one to three across domains. The OCS was administered by trained neuropsychologists and occupational therapists. The OCS is licensed through Oxford University Innovations free of charge for publicly funded research and clinical use. Further information regarding administration of the OCS is detailed in supplemental methods.

### Statistical analysis

Descriptive statistics were used to summarize the prevalence of cognitive impairment/recovery as cognitive data were limited to binarized outcome. Associations between impairments within and across timepoints were quantified using tetrachoric correlation.^20^ Sensitivity/specificity of acute impairments for predicting 6-month outcomes was calculated using receiver operating curves (ROCs).

The predictive value of early cognitive screening was quantified using hierarchical multivariable regression. Three models were produced progressing in terms of cognitive detail with cognitive performance at follow-up considered as the dependent variable. Model 1 examined the relationship between proportion of cognitive subtests impaired acutely and at follow-up. Model 2 examined acute domain-specific function and proportion of subtests impaired at follow-up. To ensure model results are robust predictors rather than the products of model overfitting, each resultant model was cross-validated. Each full model’s resultant significant covariates were iteratively (*n* = 1000) fitted to a random data subset (70%) and tested in the remaining 30% (testing data). If models yield above-chance fits in testing data, this suggests that the identified covariates are robust predictors of patient outcomes.

Model 3 examined each domain individually at acute and follow-up. Demographic/clinical factors were entered in the first block, and acute cognition was added in the second block. Demographic covariates included age, sex, and years of education. Clinical variables included stroke severity (the National Institute of Health Stroke Scale (NIHSS)), lesion volume, recurrent stroke, atrial fibrillation, hypertension, diabetes, smoking, and days from stroke onset to cognitive assessment. Lesion masks were constructed using a standard protocol.^21^ Missing NIHSS data were amended through multiple imputation. Analyses with p-values less than 0.05 were considered significant (Bonferroni-corrected where appropriate). All analyses were performed in R version 4.0.5.

## Results

Demographics and clinical characteristics of the cohort are outlined in Table 1. The OCS was administered a mean of 4.39 (4.46 SD) days and again at a mean 6.65 (1.06 SD) months after stroke. Patient demographics did not statistically differ between those who were and were not reassessed; however, the attrition group comprised more severe stroke and cognitive impairments (Supplemental Tables 1 and 2).

**Table 1.** Cohort demographics and clinical characteristics.

	*N* = 430
Age at stroke, mean/SD	73.86/12.51
Sex (female), *n* (%)	200 (46.51)
Education years, mean/SD	12.25/3.55
Handedness—right, *n* (%)	374 (86.98)
Stroke subtype, *n* (%)	
Ischemic	362 (84.19)
Hemorrhagic	65 (15.12)
Mixed	3 (1.00)
Lesion side, *n* (%)	
Left	153 (35.58)
Right	168 (39.07)
Bilateral	34 (7.91)
Undetermined	75 (17.44)
Major vascular territory,^a^ *n* (%)	
Anterior	39 (9.07)
Middle	177 (41.16)
Posterior	59 (13.72)
Vertebrobasilar	55 (12.79)
Multifocal	8 (1.86)
Lacunar	18 (4.19)
Undetermined	74 (17.21)
First-ever stroke, *n* (%)	292 (67.91)
NIHSS,^b^ median/IQR	5/2–10
Modified Rankin Scale,^c^ median/IQR	1/0–2
Barthel index,^d^ median/IQR	15/9–19
Independent at admission, *n* (%)	376 (87.44)
Dependent (care required),^e^ *n* (%)	54 (12.56)
Comorbidities, *n* (%)	
CCI low (0–1)	268 (62.33)
CCI high (⩾2)	162 (37.67)
Atrial fibrillation	109 (25.35)
Hypertension	261 (60.70)
Diabetes mellitus	83 (19.30)
Smoking, *n* (%)	
Current	44 (10.23)
Past	34 (7.91)
Never	352 (81.86)
Days from stroke to T1 assessment, mean (SD)	4.38 (4.46)
Length of stay in hospital, mean days (SD)	11.64 (11.44)

IQR: interquartile range; CCI: Charlson Comorbidity Index; NIHSS: National Institute of Health Stroke Scale.

CCI scored based on ICD-10 scoring scheme through hospital records. Population data inclusive of these patients (Oxford Vascular Study)^22^ indicate the predominant ethnicity (>90%) was White British.

a Vascular supply is a crude characterization of primary territory affected.

b NIHSS recorded for *n* = 320 (74%) due to not being recorded before 2014.

c Premorbid modified Rankin Scale (mRS) reported for *n* = 248 (58%).

d Barthel index was reported for *n* = 141 (33%).

e Dependence was categorized as requiring formal support.

### Cognitive impairments are highly prevalent acutely and at follow-up

**Figure 2.**
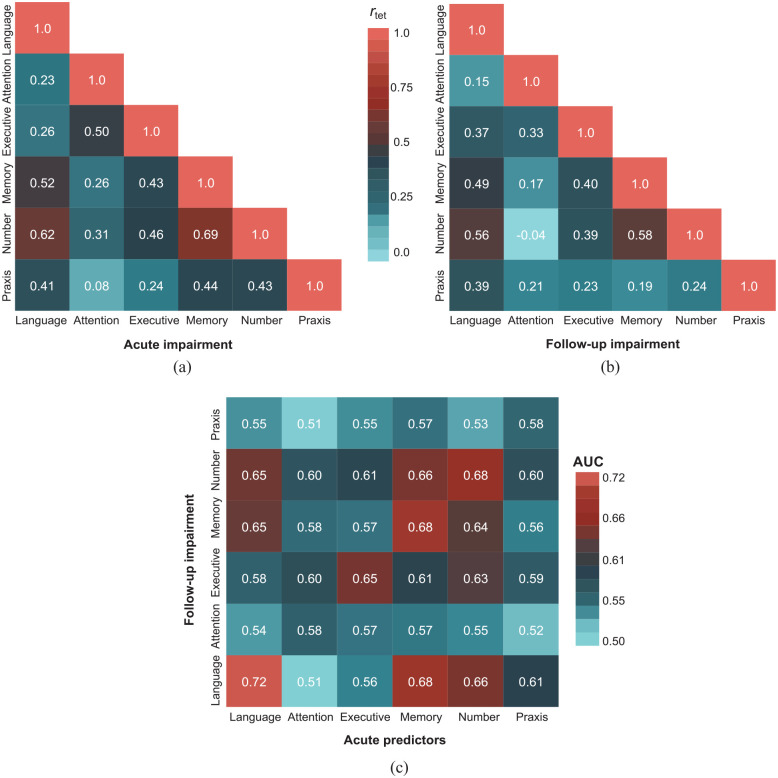
Association between acute and follow-up domain impairments and acute impairments predictive of follow-up impairments: (a) Associations between acute domain impairments. (b) Associations between follow-up impairments. (c) Acute impairments predicting follow-up impairments. For (a and b), tetrachoric correlation coefficient is shown with color indicating correlation coefficients. For (c), color indicates AUC; acute: x-axis, follow-up: y-axis.

Prevalence of post-stroke cognitive impairments acutely and at 6 months is outlined in Table 2. Overall, 423 (98.4%) experienced at least one subtest impairment acutely (*n* = 316; 73.7% exhibited multi-domain deficits), and 293 (68.1%) were impaired at follow-up (*n* = 197; 45.8% multi-domain). Impairments were prevalent within all domains at both timepoints, ranging from 112 (26.7%) in praxis to 183 (46.8%) in attention acutely, and 79 (19.6%) in praxis to 140 (32.6%) in language at follow-up. Associations between acute and 6-month paired domain impairments were strongest between memory and number processing (acute r_tet_ = 0.69, *p* < 0.001; follow-up r_tet_ = 0.58, *p* < 0.001), as well as language and number processing (acute r_tet_ = 0.62, *p* < 0.001; follow-up r_tet_ = 0.56, *p* < 0.01) (Figure 2).

**Table 2.** Prevalence of post-stroke cognitive impairment acutely and at follow-up.

Domain	Assessed acute, *n*	Acute impaired, *n* (%)	Assessed follow-up, *n*	Follow-up impaired, *n* (%)	Assessed at both timepoints, *n* (%)^a^
Language	**429**	**194 (45.2)**	**430**	**140 (32.6)**	397 (92.3)
Picture naming	429	138 (32.2)	428	78 (18.2)	428 (99.5)
Semantic understanding	427	43 (10.1)	412	16 (3.9)	410 (95.3)
Sentence reading	419	136 (32.5)	422	88 (20.9)	414 (96.3)
Spatial attention	**391**	**183 (46.8)**	**415**	**129 (31.1)**	381 (88.6)
Egocentric attention	391	124 (31.7)	415	57 (13.7)	381 (88.6)
Allocentric attention	391	108 (27.6)	414	101 (24.4)	382 (88.8)
Executive function	**379**	**111 (29.3)**	**405**	**98 (24.2)**	361 (84.0)
Memory	**429**	**171 (39.9)**	**430**	**137 (31.9)**	377 (97.7)
Orientation	427	90 (21.1)	426	72 (16.9)	423 (98.4)
Verbal memory	427	107 (25.1)	417	78 (18.7)	414 (96.3)
Episodic memory	391	79 (20.2)	416	53 (12.7)	379 (88.1)
Number processing	**427**	**178 (41.7)**	**418**	**85 (20.3)**	394 (91.6)
Calculations	427	73 (17.1)	417	39 (9.4)	414 (96.3)
Writing	411	160 (38.9)	407	64 (15.7)	395 (91.9)
Praxis	**419**	**112 (26.7)**	**403**	**79 (19.6)**	394 (91.6)
Any domain	430	423 (98.4)	430	293 (68.1)	–
Multi-domain	429	316 (73.7)	430	197 (45.8)	–
Single domain	430	107 (24.9)	430	96 (22.3)	–

Domain impairment prevalence at acute and 6-month assessments. Any domain: an impairment on any subtest in any domain; multi-domain: impairment in ⩾ 1 domain. Supplemental Table 2 summarizes patients not followed-up at 6 months.

a Prevalence of impairment on each subtest for only patients who were assessed at both acute and 6 months for each subtest is detailed in Supplemental Table 3b. All domains were assessed at both timepoints in *n* = 322/430 (74.8%).

### Recovery proportion varies across domains

**Figure 3.**
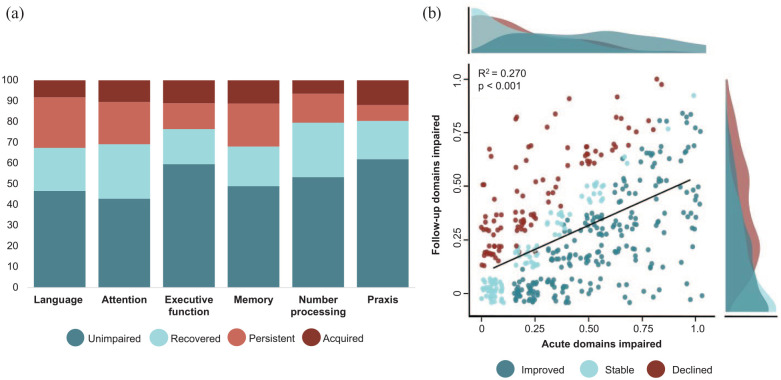
Proportion of change in impairment across timepoints. (a) Unimpaired: proportion of individuals who remained unimpaired, recovered: were impaired, but recovered by follow-up, persistent: remained impaired, and acquired (red): were unimpaired initially, but were impaired at follow-up. Across all domains, 52.2% were unimpaired, 21.3% recovered, 16.6% had persistent impairment, and 9.9% acquired impairment. (b) Improved: fewer domains impaired at follow-up, stable: no change, and declined: more domains impaired at follow-up. Solid line represents the regression with the considered clinical/demographic covariates.

Prevalence of impairment decreased across all domains from acute to follow-up (Figure 3). Recovery proportion was highest in praxis (70.9%), followed by number processing (65.3%), executive function (57.5%), attention (56.2%), memory (48.0%), and language (45.9%). While most acutely unimpaired patients remained unimpaired, newly acquired impairments were found in each domain, predominantly in attention (19.6%) and memory (18.6%) (Figure 3; Supplemental Table 3). From acute to 6 months, the strongest associations between impairments were between language (both timepoints) (r_tet_ = 0.56, *p* < 0.01), memory (both timepoints) (r_tet_ = 0.54, *p* < 0.001), and memory (acute)/language (follow-up) (r_tet_ = 0.55, *p* < 0.001) (Figure 2(c); ROC analysis shown with Supplemental Figure 1).

### Cognitive variables outperform clinical variables in predicting cognitive outcomes

Hierarchical regression showed the base model (Block 1) of common risk factors to significantly predict proportion of subtests impaired at follow-up (*F*(11, 333) = 4.69, *p* < 0.0001, adj *R*^2^ = 0.105) (Table 3). In this model, age (β = 0.005, *p* < 0.0001), education (β = −0.009, *p* = 0.012), smoking (β = 0.072, *p* = 0.026), and lesion volume (β = 0.000, *p* = 0.004) were significantly associated, though only age and lesion volume remained significant after correction (Bonferroni-corrected alpha level = 0.005). Model 1 (Block 1) remained robust to cross-validation with 86.5% of tested models yielding above-chance predictions of the proportion of impaired cognitive domain subtests at 6 months within the new testing data (average testing model *R*^2^ = 0.083 (SD = 0.04), range = 3.97 × 10^−7^–0.276).

**Table 3.** Regression results for clinical/demographic and acute cognitive factors predictive of follow-up impairment.

	Proportion of OCS subtests impaired at 6 months		
Block 1: Demographic/clinical factors	β (SE)	*t*	*p*
Age	0.094 (0.001)	4.817	0.000*^a^
Sex (male)	−0.006 (0.026)	−0.222	0.825
Education	−0.009 (0.004)	−2.527	0.012*
Atrial fibrillation	−0.025 (0.030)	−0.847	0.398
Hypertension	−0.031 (0.026)	−1.182	0.238
Diabetes	0.041 (0.031)	1.325	0.186
Smoking	0.073 (0.032)	2.241	0.026*
NIHSS	0.004 (0.003)	1.372	0.171
Recurrent stroke	0.045(0.032)	1.646	0.107
Days to assessment	0.001 (0.003)	0.476	0.635
Lesion volume	<0.001 (<0.001)	2.923	0.004*
*R*^2^ = 0.134, Adjusted *R*^2^ = 0.105, *F* = 4.693, 11 and 333 *df, p* < 0.0001			
Block 2 (Model 1): Proportion of acute subtests impaired	β (SE)	*t*	*p*
Age	0.005 (0.001)	4.674	0.000*^a^
Sex (male)	−0.004 (0.023)	−0.197	0.844
Education years	−0.005 (0.003)	−1.527	0.128
Atrial fibrillation	−0.032 (0.026)	−1.223	0.222
Hypertension	−0.033 (0.023)	−1.452	0.147
Diabetes	−0.006 (0.028)	−0.213	0. 831
Smoking	0.006 (0.029)	2.066	0.040*
NIHSS	−0.000 (0.002)	−0.134	0.893
Recurrent stroke	0.045 (0.024)	1.862	0.064
Days to assessment	0.002 (0.003)	0.786	0.433
Lesion volume	<0.001 (<0.001)	1.132	0.2585
Severity of acute cognitive impairment	0.403 (0.042)	9.616	0.000*^a^
*R*^2^ = 0.323, Adjusted *R*^2^ = 0.298, *F* = 13.190, 12 and 332 *df, p* < 0.0001			
Block 2 (Model 2): Acute domain-specific impairment	β (SE)	*t*	*p*
Age	0.004 (0.001)	4.266	0.000*^a^
Sex (male)	0.009 (0.023)	0.399	0.690
Education years	−0.005 (0.003)	−1.475	0.141
Atrial fibrillation	−0.027 (0.026)	−1.040	0.299
Block 2 (Model 2): Acute domain-specific impairment	β (SE)	*t*	*p*
Hypertension	−0.027 (0.023)	−1.182	0.238
Diabetes	−0.003 (0.029)	−0.094	0.925
Smoking	0.076 (0.029)	2.618	0.009*
NIHSS	0.001 (0.002)	0.529	0.597
Recurrent stroke	0.039 (0.024)	1.619	0.107
Days to assessment	0.000 (0.003)	0.155	0.877
Lesion volume	0.000 (0.000)	0.715	0.475
Language	0.095 (0.027)	4.303	0.000*^a^
Attention	0.011 (0.024)	0.446	0.656
Executive	0.065 (0.027)	2.376	0.018*
Memory	0.116 (0.027)	4.303	0.000*^a^
Number	0.041 (0.028)	1.477	0.141
Praxis	0.084 (0.028)	3.042	0.002*^a^
*R*^2^ = 0.345, Adjusted *R*^2^ = 0.309, *F* = 9.612, 167 and 310 *df, p* < 0.0001			

OCS: Oxford Cognitive Screen; NIHSS: National Institute of Health Stroke Scale.

a Remained significant after Bonferroni correction for multiple comparisons.

* Significance *p* < 0.05.

Proportion of acute subtests impaired was then added to this base model of clinical factors (Model 1, Block 2), which improved the model, adj *R*^2^ = 0.298 (*p* < 0.0001). After correction, only age (β = 0.004, *p* < 0.0001) and acute cognition (proportion of impaired subtests acutely) (β = 0.403, *p* < 0.0001) remained significant. Cross-validation indicated that the results of Model 1 (Block 2) were robust with 100% of tested models yielding above-chance fits within the new testing data (average testing model *R*^2^ = 0.271 (SD = 0.066), range = 0.025–0.513).

In Model 2 (Block 2), acute domain-specific cognitive impairments were added to the base model, which explained slightly more variance, adj *R*^2^ = 0.309 (*p* < 0.0001) (Table 3). In this expanded model, acute language (β = 0.095, *p* = 0.0002), memory (β = 0.116, *p* < 0.0001), and praxis impairments (β = 0.084, *p* = 0.003), as well as age (β = 0.004, *p* < 0.0001) remained significant after correction. Model 2 (Block 2) remained robust to cross-validation with 100% of trained models yielding above-chance predictions of the proportion of impaired cognitive domain subtests at 6 months within the testing data (average testing model *R*^2^ = 0.43 (SD = 0.07), range = 0.18–0.71). These results remained consistent within patients with first-ever stroke (see supplemental analysis).

### Acute domain impairments predict within- and cross-domain outcomes

Hierarchical regressions were conducted to identify acute factors predictive of individual domain impairments at follow-up. Each base model with only conventional predictors improved with the addition of domain-specific cognition (Supplemental Figure 2). Each follow-up impairment was best predicted by the same domain impairment acutely except attention (strongest predictor was age: OR = 1.0673, *p* = 0.0006) and number impairment, where no predictor remained significant after correction. Full results for all domain-specific regression models are presented in Supplemental Tables 4–10.

## Discussion

Nearly every patient was impaired on at least one cognitive subtest initially following stroke, while over two-thirds were impaired at follow-up. Attention and language impairments were most prevalent acutely with the addition of memory impairments at follow-up. The impairment prevalence decreased within all domains from acute to follow-up, and most participants who were unimpaired acutely remained unimpaired at follow-up. However, newly acquired impairments were found across all domains at follow-up. The severity of acute domain-specific cognitive impairment identified with the OCS was the strongest predictor of follow-up cognitive function, over and above conventional demographic and clinical factors. Acute impairments in memory, language, and praxis were particularly important in predicting the severity of follow-up cognitive impairment.

The high impairment prevalence is consistent with studies reporting post-stroke cognitive impairment prevalence acutely (ranging 49–92%)^1,14,23–26^ and at follow-up (41–57%).^26–28^ Our findings of higher prevalence at both timepoints are likely due to this study’s representative sample including potential pre-stroke cognitive decline, recurrent strokes, severe aphasia,^29^ and the timing of acute assessment (average 4 days post-stroke). In addition, the OCS has higher sensitivity to stroke-specific deficits compared to other tools.^1,9^ The increased prevalence of multi-domain impairments found in this cohort is in line with Nys et al.^18^ who reported that impaired patients averaged three domain deficits using a neuropsychological battery. Similarly, Jokinen et al.^30^ found that 50% were impaired in three or more domains. The frequency of multi-domain impairments is reflected in clinical stroke guidance emphasizing the need for multi-domain screening.^3^ Furthermore, global screens that fail to assess stroke-specific deficits, such as visuospatial neglect, may omit information valuable to rehabilitation planning.^1,9^

The frequency of domain-specific deficits reported by previous studies varies. For example, both Hurford et al.^25^ and Leśniak et al.^24^ found attention to be the most commonly affected domain, though their attention measures included different executive demands. Different measures and domain definitions may explain variability across studies. In our cohort, attention and language were the most frequently impaired domains, which are hallmark deficits of lateralized stroke.^31,32^ A high prevalence of spatial attention impairments was also found in previous studies.^33^ Both neglect types were prevalent in left- and right-sided stroke, highlighting that allocentric and right-sided neglect are not uncommon.^34^ Comparatively high language impairment may be explained by our inclusion of aphasic patients. Aphasia is present in 30% of acute stroke patients^35^and excluding these patients risks biasing post-stroke cognitive profiles. In addition, though number processing, memory, executive function, and praxis deficits were less frequent, impaired subtests still occurred in 27–42% acutely and 20–32% at follow-up. This is consistent with the studies highlighting executive function, processing speed, and episodic memory as more commonly reported deficits months after stroke.^23,25,30^ Overall, cognitive impairments occur in multiple domains, frequently affecting complex abilities in which attention and language have a major role.

Impairment prevalence decreased across all domains from acute to follow-up, aligning with previous research.^18,19,25^ The highest proportion of recovery was in praxis and number processing, while language and memory impairments were most persistent. This aligns with the study by Hurford et al.^25^ where memory deficits did not change between assessments but contradicts the study by Turunen et al.,^26^ which found the greatest rates of recovery from baseline to 6 months in executive functions and visual memory. Persistent and acquired memory deficits could be due to memory being associated with pre-stroke neurodegeneration, which would not be expected to change over a relatively short period. Similarly, persistent language deficits were consistent with reports of long-term language impairments at 1-year post-stroke,^33^ and of language recovery decreasing beyond 6 months post-stroke.^36^ Another important finding is the proportion of acquired impairments observed, ranging 11–20%, and consistent with Nys et al.^19^ who reported new impairments at 6-month follow-up in all five domains assessed. The presence of varying domain outcomes in this sample mirrors previous varying overall trends toward recovery, stability, and decline.^37,38^ In general, the risk of post-stroke cognitive decline is dependent on the combination of pre-existing cerebral vulnerability/reserve and the impact of stroke.^39^

The key finding of this study is that routine early domain-specific screening with the OCS can predict 6-month cognitive function and provide domain-specific profiling. Critically, the aim of this study was not to identify an optimal predictive model, but to determine if acute cognition could provide useful prognostic information. Acute cognitive functioning was strongly associated with both severity of cognitive impairment at follow-up and domain-specific impairment, explaining ~30% more outcome variance than conventional demographic and clinical factors. More specifically, memory, language, and praxis deficits acutely were highly significant predictors of the severity of cognitive impairment at follow-up. Age emerged as the most consistent demographic risk factor for severity of cognitive impairment at follow-up and was the strongest predictor of a 6-month attention impairment. Collectively, these findings indicate that both age and acute domain-specific cognitive function as measured by the OCS are important when considering neurorehabilitation and cognitive trajectories post-stroke. These findings support the current recommendation for routine early domain-specific cognitive screening post-stroke and demonstrate the prognostic value of early cognitive markers, both of which have been highlighted as priorities for stroke research.^4,6,40^

This study has strengths, including a representative sample, a balance of minor and major stroke, and longitudinal domain-specific cognitive screening. However, there are also limitations. Though efforts were made to maximize inclusivity, all studies requiring a sufficient comprehension to provide (witnessed) informed consent will exclude those with most severe stroke and the poorest cognitive outcomes. A proportion of attrition was due to death (19%), again representing a potential source of bias toward underrepresentation of more severe stroke. Nevertheless, attrition in a representative stroke sample is generally unavoidable and a relatively large sample size was retained.

In summary, early domain-specific cognitive profiling with the OCS provides valuable prognostic information with respect to longer-term cognitive functioning. The OCS is currently used routinely in clinical settings and these findings suggest it could be employed for prognostic purposes within a stroke care pathway, prompting further follow-up with a more detailed neuropsychological battery. Cognitive impairment was highly prevalent initially after stroke, though after 6 months, prevalence of impairment had decreased across all domains demonstrating a general trend toward recovery. However, persistent impairments were also found across all domains, particularly memory and language deficits. Each post-stroke cognitive profile is unique and highlighting different strengths and weaknesses in performance early allows for more accurate information to be communicated to the patient,^22^ more tailored discharge care packages and appropriate allocation of rehabilitation resources.

## Supplemental Material

sj-docx-1-wso-10.1177_17474930231205787 – Supplemental material for Domain-specific cognitive impairment 6 months after stroke: The value of early cognitive screening Supplemental material, sj-docx-1-wso-10.1177_17474930231205787 for Domain-specific cognitive impairment 6 months after stroke: The value of early cognitive screening by Elise T Milosevich, Margaret J Moore, Sarah T Pendlebury and Nele Demeyere in International Journal of Stroke
